# From cognitive need to problematic use: a chained mediation path moderated by academic stress and AI literacy

**DOI:** 10.3389/fpsyg.2026.1767454

**Published:** 2026-03-16

**Authors:** Yong Kong, Tongqiang Dong, Ziyi Yang, Yu Fang, Ning Chen

**Affiliations:** School of Communication, Qufu Normal University, Rizhao, China

**Keywords:** academic stress, AI literacy, Generative AI, I-PACE model, moderated mediation, need for cognition, problematic use

## Abstract

**Introduction:**

The rapid adoption of Generative AI (GenAI) in higher education raises concerns about psychological dependency. Grounded in the I-PACE model, this study investigates how lower need for cognition (NFC) is associated with problematic use via positive affect and Avoidance-Oriented GenAI Motivation, moderated by academic stress and AI literacy.

**Methods:**

To test the hypothesized model, we employed a two-wave, time-lagged survey design with a sample of university students (*N* = 452). Data were analyzed using structural equation modeling (SEM) to assess the serial mediation effects and the moderated mediation dynamics.

**Results:**

Analysis confirmed a serial mediation chain: lower NFC predicted stronger positive affect, increasing avoidance motivation and subsequent problematic use. This pathway was significantly amplified by high academic stress but attenuated by high AI literacy, which neutralized dependency risks.

**Discussion:**

Findings extend the I-PACE model to instrumental technologies, identifying a specific “cognitive relief to avoidance” mechanism. The study highlights AI literacy as a vital protective resource, suggesting educators should prioritize fostering critical digital competencies over prohibitive policies to mitigate reliance risks.

## 1 Introduction

The advent of powerful Generative Artificial Intelligence (GenAI), exemplified by models like ChatGPT, marks a technological inflection point with profound implications for higher education (Dwivedi et al., 2023). These tools offer unprecedented capabilities for information synthesis, content creation, and problem-solving, promising to enhance learning efficiency and democratize access to knowledge (Moorhouse, 2024). However, alongside this vast potential, a growing chorus of concern has emerged among educators and scholars regarding the darker side of human-AI interaction. The very power and convenience that make GenAI so attractive may foster intellectual passivity, erode critical thinking skills, and ultimately lead to a form of problematic psychological dependency (Routledge, 2025; Hwang and Wu, 2025).

Before exploring these risks, it is crucial to theoretically distinguish between “problematic use” and clinical “addiction” or “dependency.” In this study, we adopt the Cognitive-Behavioral Model of Pathological Internet Use but frame Problematic GenAI Use not as a clinical pathology, but as a maladaptive functional coping style. Unlike addiction, which implies severe withdrawal symptoms and physiological tolerance, problematic use in our context refers to an over-reliance on GenAI that interferes with daily functioning and academic integrity. We posit that students may develop a psychological reliance on GenAI to regulate negative affect or avoid cognitive effort, which creates “problematic” outcomes without necessarily meeting the threshold of a clinical addiction disorder.

It is also vital to distinguish the scope of this study from the burgeoning literature on academic integrity. While recent scholarship has extensively documented how GenAI facilitates plagiarism and contract cheating (Perkins et al., 2024), our focus diverges from misconduct to the psychological mechanism of dependency. We argue that problematic use is a distinct phenomenon: a student may compulsively rely on GenAI to alleviate cognitive load without necessarily violating academic integrity policies (e.g., using it for approved brainstorming but becoming unable to function without it). This distinction aligns with research on “functional impairments” in technology use, where the harm lies in the erosion of autonomous capability rather than rule-breaking *per se*.

While many opinion pieces and early exploratory studies have highlighted these risks, the empirical literature is still in its infancy. A significant gap exists in our understanding of the psychological mechanisms associated with the transition from a useful tool into an object of such maladaptive reliance. It is no longer sufficient to ask if GenAI use can become problematic; we must now investigate the associations between these factors and, critically, under what conditions these relationships are most pronounced. Are certain individuals more vulnerable? What specific affective and cognitive pathways are associated with the transition from initial use to a state of over-reliance? Furthermore, are there contextual or personal factors that exacerbate or mitigate this risk? Answering these questions is crucial for developing evidence-based, targeted educational strategies and for designing AI systems that support, rather than subvert, genuine learning.

To address this gap, we turn to the Interaction of Person-Affect-Cognition-Execution (I-PACE) model, a comprehensive theoretical framework for understanding the development and maintenance of problematic behaviors (Pian et al., 2024). Recent updates to the model continue to support its application to a wide range of specific internet-use disorders (Kumalaratih and Margono, 2023). The I-PACE model provides a dynamic, process-oriented perspective that integrates predisposing individual characteristics, specific affective and cognitive responses, and resulting behavioral patterns. While traditionally applied to hedonic technologies like social media and online gaming, we argue its core logic offers a powerful lens for examining problematic use of instrumental technologies like GenAI. The psychological rewards of using GenAI—such as feelings of efficiency and relief from cognitive strain—can be as reinforcing as hedonic rewards, potentially setting in motion a similar feedback loop of dependency (Kim, 2024). Our study leverages this framework to systematically map the psychological pathway from initial use to over-reliance.

Our theoretical model synthesizes several established research streams to map this trajectory. Specifically, we anchor the “Person” component in Cacioppo and Petty's (1982) Need for Cognition (NFC), a trait long associated with information processing depth but under-explored in AI contexts. The “Cognition” component integrates the concept of avoidance learning motivation (Elliot and McGregor, 2001), positioning it as a maladaptive response to the “cognitive offloading” afforded by AI. Furthermore, we draw on the transactional theory of stress to frame academic stress as a situational amplifier, and extend recent digital literacy frameworks (Ng et al., 2021; Long and Magerko, 2020) to position AI literacy not just as a skill, but as a psychological buffer. By integrating these constructs into the I-PACE framework—traditionally used for hedonic behaviors like gaming—we extend its application to the novel domain of instrumental AI dependency.

Within the I-PACE framework, a key predisposing factor is an individual's cognitive style. Whereas some research has focused on “inert thinking” as a risk factor (Ye et al., 2025), we propose examining its conceptual counterpart: Need for Cognition (NFC). NFC refers to an individual's intrinsic motivation to engage in and enjoy effortful cognitive endeavors (Cacioppo and Petty, 1982). Individuals high in NFC are “chronic cognizers” who relish intellectual challenges, while those low in NFC tend to be “cognitive misers,” preferring to rely on heuristics and simple cues to conserve mental energy. We hypothesize that a low NFC represents a critical vulnerability. For these individuals, the ability of GenAI to provide instant, well-structured answers may be particularly gratifying, serving as a welcome escape from the discomfort of cognitive effort.

This gratification manifests as a positive affective experience—the first crucial link in our proposed mechanism. When using GenAI alleviates the stress of a difficult task, it can produce feelings of satisfaction, relief, and competence. According to the I-PACE model, such positive affective responses act as powerful reinforcers, strengthening the user's tendency to turn to the technology in the future (Salah et al., 2024).

However, we propose that this affective reinforcement has a further, more insidious cognitive consequence: it fosters avoidance-oriented genAI motivation. This construct, drawn from achievement goal theory, describes a motivation driven by the desire to avoid struggle, failure, or the appearance of incompetence (Elliot and McGregor, 2001). As students repeatedly experience the ease and success afforded by GenAI, they may learn to devalue intellectual struggle and actively seek to circumvent challenging learning tasks. The tool ceases to be a learning aid and instead becomes an escape route.

This leads to our core mediational hypothesis: a lower Need for Cognition is the starting point of a psychological cascade that predicts problematic GenAI use through the sequential pathway of (1) an enhanced positive affective experience, which in turn fosters (2) a stronger avoidance-oriented genAI motivation. However, this pathway may not be deterministic. The link between a motivation to avoid effort and the actual problematic behavior is likely contingent on other factors. This brings us to the question of boundary conditions.

We propose that the progression from an avoidant motivation to problematic use is not uniform across all students or situations. Drawing on stress-and-coping theories, we identify key factors that may moderate this crucial final step. For instance, high levels of perceived academic stress may act as an accelerator. When students feel overwhelmed by course demands, an existing motivation to avoid difficulty is more likely to translate into a desperate reliance on AI as a coping mechanism (Lazarus and Folkman, 1984). Conversely, certain personal resources may act as a buffer. A student's AI literacy—their ability to critically evaluate and judiciously use AI—could weaken this link. Students with high AI literacy, even if tempted to avoid effort, may possess the metacognitive skills to resist full dependency and use the tool in a more balanced way. Therefore, it is critical to examine these moderating influences to understand for whom the risk of dependency is most acute.

Therefore, the present study aims to test a comprehensive, moderated mediation model of how problematic GenAI use develops. Specifically, we investigate whether the sequential indirect effect of Need for Cognition on problematic use (through positive affective experience and avoidance motivation) is conditional upon levels of academic stress and AI literacy. To provide a rigorous test of this hypothesized chain and to mitigate common method bias (CMB), we employ a two-wave, time-lagged research design. By elucidating this specific psychological cascade and its boundary conditions, this study seeks to move beyond simple correlations to offer a mechanistic explanation for problematic GenAI use. The findings will not only advance the theoretical application of the I-PACE model but also provide actionable, context-sensitive insights for educators seeking to cultivate resilient, critical thinkers in the age of AI.

## 2 Theoretical models and hypotheses

### 2.1 The I-PACE model as a framework for instrumental technology use

To unpack the complex process leading to problematic use of generative AI, this study is grounded in the Interaction of Person-Affect-Cognition-Execution (I-PACE) model (Ye et al., 2025). The I-PACE model's central thesis is that problematic behaviors emerge from a dynamic interplay between predisposing individual factors (“Person”), specific affective and cognitive responses during use (“Affect-Cognition”), and the resulting maladaptive coping patterns and diminished executive control (“Execution”).

A critical theoretical step in our study is the application of this model, originally developed for hedonic technologies (e.g., gaming, social networking), to the instrumental use of GenAI. We contend that the nature of the “reward” is the key. For hedonic media, the reward is often pleasure or social connection. For instrumental tools like GenAI, the reward is utilitarian—derived from efficiency, convenience, and the successful (and effortless) completion of tasks (Roblek et al., 2021). This feeling of cognitive relief and task-related competence can be a powerful psychological reinforcer, triggering a similar reinforcement loop of craving and repeated use as that seen in hedonic media. Furthermore, this convenience can create or heighten a state of cognitive dissonance between a student's internal ideals of academic integrity (valuing effort) and their actual shortcut behaviors. To resolve this psychological discomfort, students may adopt dissonance-reduction strategies—such as rationalizing the task as “busy work”—which manifest behaviorally as avoidance and psychologically as a dependency on the tool (Seran et al., 2025).

Therefore, the I-PACE model provides an ideal theoretical scaffold for our investigation. We operationalize its core components to map the specific sequential pathway to problematic GenAI use:

The “Person” component is represented by an individual's Need for Cognition, a stable cognitive style predisposing them to either seek or avoid mental effort.

The “Affect” component is captured by the positive affective experience—the immediate feeling of satisfaction and relief when using GenAI.

The “Cognition” component is defined as avoidance-oriented genAI motivation, a maladaptive learning goal that develops as a consequence of repeated positive affective experiences.

Finally, the “Execution” component is the outcome variable, problematic use of GenAI, characterized by loss of control and preoccupation. By specifying these constructs within the I-PACE framework, we can test a precise, theory-driven model of how digital dependency on an instrumental tool develops.

### 2.2 The role of need for cognition as a predisposing factor

The I-PACE model's “Person” component highlights the role of stable individual differences. In the context of an intellectual tool like GenAI, an individual's cognitive style is a paramount vulnerability factor. This study focuses on Need for Cognition (NFC), defined as an intrinsic tendency to engage in and enjoy effortful cognitive activities (Cacioppo and Petty, 1982). NFC is a key construct in Dual Process Theory (Bonnefon and Rahwan, 2020), distinguishing individuals who habitually favor deliberate, analytical thinking (high NFC) from those who prefer heuristic, low-effort processing (low NFC), often termed “cognitive misers.”

We posit that a low NFC is a significant predisposing risk factor for developing GenAI dependency. For students who find deep thinking to be arduous or aversive, the frictionless efficiency of GenAI offers a highly attractive solution (Wang et al., 2025). The tool effectively serves as a cognitive prosthesis, allowing them to bypass the very intellectual struggle they are motivated to avoid. This alignment between a user's disposition and a technology's affordance is likely to trigger a particularly strong positive response. Therefore, we hypothesize a direct theoretical link from this stable cognitive trait to the immediate affective experience of using the tool.

H1: A lower Need for Cognition, measured at Time 1, is expected to be associated with a more positive affective experience with using Generative AI at Time 1.

### 2.3 The mediating role of affective and cognitive responses

According to the I-PACE model, the interaction between person-specific factors and technology use elicits specific affective and cognitive responses that mediate the path to problematic behavior. Our model proposes a two-stage mediation process that unfolds over time.

First, we examine positive affective experience as the initial mediator. The I-PACE model emphasizes that rewarding experiences strengthen attentional biases and cravings for technology use (Ye et al., 2025). In the context of GenAI, this reward is the feeling of relief, competence, and satisfaction that comes from quickly and easily solving a difficult academic problem. This positive affect acts as a powerful operant reinforcer. For a student with low NFC, this experience is particularly salient, validating their preference for cognitive ease. This reinforcing experience is expected not only to stem from low NFC but also to influence the student's subsequent motivation toward learning itself.

Second, we propose that this positive affective experience, in turn, cultivates avoidance-oriented genAI motivation. Drawing from achievement goal theory, avoidance motivation is characterized by a focus on evading struggle, failure, or the appearance of incompetence (Elliot and McGregor, 2001). When students learn that using GenAI is a reliable and emotionally pleasant way to circumvent intellectual challenges, they are conditioned to view effortful learning as an unnecessary and aversive state. The tool thus shifts from being a supportive scaffold to an escape route, fostering a cognitive orientation centered on avoiding academic difficulty. This shift in motivation is a critical step toward dependency, as the user's goal becomes less about learning and more about task completion with minimal effort.

Our time-lagged design allows us to model this as a developmental sequence: the initial interaction between cognitive style and affective experience (at Time 1) is hypothesized to predict the more stable learning motivation observed at a later point (Time 2).

H2: Positive affective experience (T1) is expected to mediate the relationship between Need for Cognition (T1) and avoidance-oriented genAI motivation (T2).

### 2.4 The serial pathway to problematic use of Generative AI

The culmination of this process is the development of problematic use of Generative AI, the “Execution” component in our model. We argue that problematic use is not an instantaneous outcome but the result of the reinforcing cycle described above. Both the immediate positive affect and the subsequent development of an avoidant motivational stance are expected to contribute directly to dependency. A positive experience creates a craving for the tool's immediate gratification, while avoidance motivation solidifies the tool's role as an indispensable coping mechanism for academic demands.

By integrating these components into a single, sequential model, we can test the complete psychological pathway. Our central hypothesis is that the relationship between a predisposing cognitive style (low NFC) and problematic use is not direct but is channeled through this specific affective-cognitive chain. However, we further contend that this pathway is not invariant, but is instead contingent upon specific contextual and personal factors, which we will now elaborate.

H3: Avoidance-oriented genAI motivation (T2) is expected to mediate the relationship between positive affective experience (T1) and problematic use of Generative AI (T2).

H4: A significant serial indirect relationship is expected from Need for Cognition (T1) and problematic use of Generative AI (T2) through the sequential mediation of positive affective experience (T1) and avoidance-oriented genAI motivation (T2).

### 2.5 The moderating role of academic stress and AI literacy: defining the boundary conditions

While the proposed mediation pathway outlines how dependency may develop, a more nuanced understanding requires investigating when and for whom this process is most potent. We argue that the critical link between an established avoidance learning motivation and the manifestation of problematic use is not automatic; it is subject to boundary conditions that either amplify or attenuate the risk. Grounded in prominent theories of stress, coping, and resource management, we propose two key moderators: a situational factor (perceived academic stress) and a personal resource factor (AI literacy).

#### 2.5.1 The accelerating effect of academic stress

Perceived academic stress, defined as the appraisal of academic demands as taxing or exceeding one's available resources, is a powerful catalyst for maladaptive coping behaviors (Lazarus and Folkman, 1984). According to the Conservation of Resources (COR) theory, when individuals are under stress and perceive their resources to be threatened or depleted, they are highly motivated to adopt strategies that promise to conserve remaining resources with minimal effort (Hobfoll, 1989).

In this context, a student who has already developed an avoidance-oriented genAI motivation is in a state of cognitive vulnerability. When this student is also experiencing high academic stress (e.g., facing tight deadlines, complex assignments, and high-stakes exams), GenAI presents itself as an extremely appealing, low-cost coping mechanism. The motivation to avoid struggle converges with the situational pressure to perform, making the turn to excessive, uncontrolled AI use almost irresistible. The tool is no longer just an escape from discomfort but a perceived lifeline in a sea of academic pressure. Conversely, for a student with low academic stress, even if they possess an avoidance motivation, the lack of urgent pressure may prevent this motivation from escalating into full-blown problematic use. Therefore, we hypothesize that academic stress will exacerbate the link between avoidance motivation and problematic dependency.

H5a: Perceived academic stress is expected to moderate the relationship between avoidance-oriented genAI motivation (T2) and problematic use of Generative AI (T2), such that the association is stronger for students experiencing higher levels of academic stress.

#### 2.5.2 The buffering effect of AI literacy

While situational pressures can amplify risk, personal resources can serve as a protective buffer. We propose that AI literacy is one such critical resource. AI literacy is more than just technical proficiency; it is a multi-dimensional construct encompassing the ability to critically evaluate AI-generated content, understand its underlying mechanisms and limitations, and apply it ethically and effectively in context (Long and Magerko, 2020). Recent research confirms that higher AI literacy is associated with a greater intention to use GenAI constructively for learning, mediated by its perceived value (Al-Abdullatif and Alsubaie, 2024).

For students with a high level of AI literacy, their relationship with the technology is more sophisticated. Even if they harbor an avoidance motivation, their advanced understanding allows them to maintain psychological distance and control. They are more likely to recognize when AI-generated content is superficial, to question its accuracy, and to understand that over-reliance will ultimately undermine their learning goals. They possess the metacognitive “brakes” to stop themselves from sliding down the slippery slope from use to misuse. In contrast, students with low AI literacy lack this critical framework. They may view GenAI as an infallible “black box” oracle. For them, an avoidance motivation is more likely to translate directly into credulous, excessive, and ultimately problematic use, as they lack the skills and awareness to self-regulate. Thus, AI literacy should weaken the link between avoidance motivation and problematic use.

H5b: AI literacy is expected to moderate the relationship between avoidance-oriented genAI motivation (T2) and problematic use of Generative AI (T2), such that the association is weaker for students with higher levels of AI literacy.

### 2.6 The full moderated mediation model

**Figure 1:**
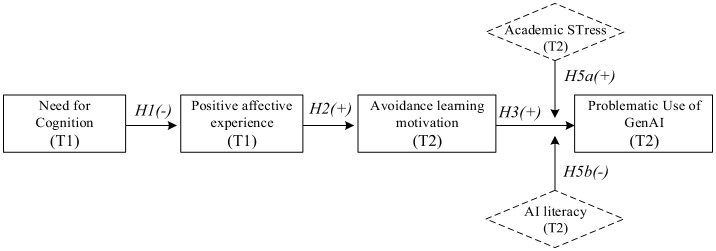
The hypothesized moderated serial mediation model of problematic Generative AI use. This conceptual model illustrates the proposed psychological pathway from predisposing cognitive style to behavioral dependency based on the I-PACE framework. Pathways: The model posits that lower need for cognition (T1) predicts higher problematic use (T2) through a two-step serial mediation: first, by increasing positive affective experience (T1), which subsequently fosters avoidance-oriented GenAI motivation (T2). moderation: the final link between avoidance motivation and problematic use is hypothesized to be conditional. Perceived academic stress (T2) is expected to amplify this relationship (risk factor), while AI literacy (T2) is expected to attenuate it (protective factor). Solid arrows represent hypothesized directional associations; dashed arrows represent moderating effects. T1, Time 1; T2, Time 2 (4-week lag).

By integrating these moderators, our study moves beyond a simple mediation model to test a more comprehensive moderated mediation model. This model posits that the entire indirect effect of our proposed mediation chain is conditional. Specifically, we test the “second stage” moderated mediation, where the strength of the sequential indirect pathway from Need for Cognition to problematic use (via positive affect and avoidance motivation) is dependent on the levels of academic stress and AI literacy. This allows us to answer the ultimate question: Under what conditions does a low-effort cognitive style most potently lead to digital dependency? The integrated conceptual model is illustrated in Figure 1.

H6a: Perceived academic stress is expected to moderate the serial indirect effect specified in H4, such that the indirect effect is stronger at higher levels of academic stress.

H6b: AI literacy is expected to moderate the serial indirect effect specified in H4, such that the indirect effect is weaker at higher levels of AI literacy.

## 3 Method

This section outlines the methodological approach employed to test the hypothesized moderated mediation model. It details the research design, participant recruitment, measurement instruments, data analysis procedures, and ethical considerations.

### 3.1 Participants and procedure

This study employed a two-wave, time-lagged survey design to test the proposed sequential mediation model and to mitigate the risk of common method bias inherent in cross-sectional research. The target population consisted of undergraduate and graduate students from a large, comprehensive university in China. Participants were recruited through university-wide email lists and course announcements, with participation being voluntary. A small course credit or a gift card lottery entry was offered as an incentive for completing both waves of the survey.

Data collection occurred in two phases, separated by a 4-week interval. This time lag was chosen because the first 4 weeks of a semester typically mark the transition from initial course orientation to the onset of significant academic workload. Crucially, psychological research on habit formation suggests that the novelty effect of new technologies typically fades within the first month. This 4-week window allows us to capture the critical shift where initial curiosity crystallizes into stable, habituated patterns of avoidance or constructive use before they become deeply entrenched. This interval captures the critical window where initial affective impressions of GenAI solidify into stable coping strategies under increasing pressure, while remaining short enough to minimize participant attrition (Cole and Maxwell, 2003).

Time 1 (T1): The initial online survey was administered at the beginning of the academic semester. Participants provided demographic information and completed the scales for Need for Cognition and Positive Affective Experience with Generative AI. A total of 580 students completed the T1 survey.

Time 2 (T2): 4 weeks later, the 580 participants from T1 were invited via email to complete the second survey. This survey included the scales for Avoidance-Oriented GenAI Motivation, Perceived Academic Stress, AI Literacy, and Problematic Use of Generative AI. The moderators and the final outcome variable were measured at T2 to reflect a more proximal influence on problematic behaviors.

After data collection, responses were matched using a unique, self-generated identifier to ensure anonymity. Questionnaires with significant missing data or from participants who failed an attention-check item were excluded. This resulted in a final matched sample of 452 students (77.9% retention rate), which was used for the main analysis.

The final sample consisted of 262 females (58.0%) and 190 males (42.0%). The age of the participants ranged from 18 to 28 years, with a mean age of 21.4 years (*SD* = 2.8). The sample included 315 undergraduate students (69.7%) and 137 graduate students (30.3%). All participants reported using Generative AI tools for academic purposes at least once a week.

### 3.2 Measures

All constructs were measured using established scales from the literature, adapted where necessary to the context of Generative AI. A 5-point Likert scale (1 = “Strongly Disagree” to 5 = “Strongly Agree”) was used for all items. The original English scales were translated into Mandarin Chinese and then back-translated by two bilingual researchers to ensure semantic equivalence. A pilot test with 30 students confirmed the clarity and appropriateness of the items.

Need for Cognition (T1). This was measured using the 18-item short form of the Need for Cognition Scale (Cacioppo et al., 1984). This scale assesses an individual's tendency to engage in and enjoy effortful thinking. A sample item is, “I prefer complex to simple problems.” The scale demonstrated excellent internal consistency in the current study (α = 0.91).

Positive Affective Experience (T1). This was measured using a 5-item scale adapted from the gratification subscale of the Internet Use Gratification Scale (IUGS; Wei et al., 2024). The items were modified to specifically capture the positive feelings associated with using GenAI for academic tasks. A sample item is, “Using Generative AI to solve a difficult assignment makes me feel a sense of relief and satisfaction.” The internal consistency for this scale was high (α = 0.88).

Avoidance-Oriented GenAI Motivation (T2). To capture the specific instrumental motivation behind GenAI use, we adapted the performance-avoidance goals subscale of the Achievement Goal Questionnaire-Revised (AGQ-R; Elliot and Murayama, 2008). The items were contextualized to reference GenAI explicitly (e.g., “My main goal when using GenAI for my studies is to avoid difficult tasks as much as possible”). While this wording references GenAI behaviors, we draw a strict conceptual distinction between the motivation to avoid effort and the pathology of problematic use. This scale measures the strategic intent to minimize cognitive load (a functional goal), whereas the Problematic Use scale measures the loss of control and compulsive dependency (a dysfunctional outcome). CFA results (see Results section) confirm that these constructs are empirically distinct despite their contextual proximity. The full list of items is provided in Appendix A.

Problematic Use of Generative AI (T2). This was measured using a 7-item scale adapted from the Problematic Internet Use Questionnaire (PIUQ; Demetrovics et al., 2008), focusing on core components like loss of control and preoccupation. Items were contextualized for GenAI. A sample item is, “I find it difficult to stop using Generative AI even when I know I should focus on other things.” The scale demonstrated excellent internal consistency (α = 0.90).

Perceived Academic Stress (T2). This was measured using a 5-item scale adapted from the Perceived Stress Scale (PSS; Cohen et al., 1983) and contextualized for the academic environment. Participants rated how often they felt certain ways during the past month. A sample item is, “In the last month, how often have you felt that you were unable to control the important things in your academic life?” This scale demonstrated good internal consistency (α = 0.87).

AI Literacy (T2). This construct was measured using a 7-item scale developed based on the conceptual framework of AI literacy by Long and Magerko (2020), specifically targeting the competencies of “critical evaluation” and “ethics”. To ensure content validity, the initial item pool was subjected to an expert review process involving three experts in educational technology and psychology. The experts evaluated the items for clarity, relevance, and alignment with the theoretical framework. Based on their feedback, two items focusing solely on technical operational skills were removed to better distinguish critical literacy from mere technical proficiency, and the wording of three items was refined to emphasize evaluative judgment rather than usage frequency. Although AI literacy is theoretically multidimensional, an Exploratory Factor Analysis (EFA) on our pilot data suggested a single dominant factor explaining 62.5% of the variance. Consequently, consistent with recent empirical applications in educational settings (Wang et al., 2025), we operationalized AI literacy as a unidimensional composite score representing students' overall capability to critically and ethically engage with GenAI. Sample items include, “I am good at critically judging whether the information provided by Generative AI is accurate and reliable.” The scale demonstrated good reliability (α = 0.89). See Appendix A for the complete scale items.

### 3.3 Data analysis strategy

Data analysis was conducted using SPSS 26.0 for preliminary analysis and Mplus 8.3 for the main structural equation modeling (SEM). All SEM analyses were performed using the robust maximum likelihood estimator (MLR), which provides standard errors and a chi-square test statistic that are robust to non-normality in the data. Missing data were handled using the Full Information Maximum Likelihood (FIML) procedure within Mplus, which uses all available data and is superior to deletion methods.

The analysis proceeded in three main steps.

First, preliminary analysis was performed in SPSS, including descriptive statistics, correlation analyses, and reliability tests (Cronbach's alpha) for all scales.

Second, a Confirmatory Factor Analysis (CFA) was conducted in Mplus to establish the measurement model's validity. This step assessed the convergent and discriminant validity of the six latent constructs. Convergent validity was assessed using Average Variance Extracted (AVE > 0.50) and Composite Reliability (CR > 0.70). Discriminant validity was checked by ensuring that the square root of the AVE for each construct was greater than its correlation with any other construct (Fornell and Larcker, 1981), as shown in Table 1.

**Table 1:** Descriptive statistics and correlation matrix.

**Variable**	**Mean**	***SD***	**1**	**2**	**3**	**4**	**5**	**6**
1. Need for cognition (T1)	4.25	0.88	(0.76)					
2. Positive affective experience (T1)	3.82	0.75	−0.38^**^	(0.79)				
3. Avoidance learning motivation (T2)	3.61	0.91	−0.25^**^	0.42^**^	(0.74)			
4. Academic stress (moderator)	3.95	0.82	−0.10^*^	0.15^**^	0.22^**^	(0.78)		
5. AI literacy (moderator)	3.70	0.90	0.20^**^	−0.12^*^	−0.18^**^	−0.05	(0.78)	
6. Problematic use of GenAI (T2)	3.48	0.85	−0.28^**^	0.35^**^	0.55^**^	0.30^**^	−0.25^**^	(0.79)

*N* = 452. T1, Time 1; T2, Time 2. Values on the diagonal in parentheses are the square roots of the average variance extracted (AVE), serving as the criterion for discriminant validity. ^*^p < 0.05, ^**^p < 0.01.

Third, the primary hypotheses were tested using SEM. To test the moderation hypotheses (H5a, H5b), we created latent interaction terms in Mplus using the unconstrained approach with the XWITH command. The independent variable (Avoidance-Oriented GenAI Motivation) and the moderators (Academic Stress, AI Literacy) were mean-centered before creating the interaction terms to reduce multicollinearity and aid interpretation.

To test the moderated mediation hypotheses (H6a, H6b), we calculated the conditional indirect effects at high (+1 SD) and low (−1 SD) levels of the moderators. The significance of these conditional indirect effects was determined by examining the bias-corrected bootstrap confidence intervals (with 5,000 resamples). Furthermore, the significance of the overall moderated mediation was tested by computing the “index of moderated mediation” (Hayes, 2015). A 95% confidence interval for this index that does not contain zero indicates that the indirect effect significantly varies across different levels of the moderator.

Model fit was evaluated using a range of indices and established cutoff criteria: the chi-square to degrees of freedom ratio (χ^2^/df < 3), the Comparative Fit Index (CFI > 0.95), the Tucker-Lewis Index (TLI > 0.95), the Root Mean Square Error of Approximation (RMSEA < 0.06), and the Standardized Root Mean Square Residual (SRMR < 0.08) (Hu and Bentler, 1999).

Control for Common Method Bias: Given the reliance on self-report measures, particularly for variables assessed concurrently at Time 2, common method bias (CMB) remains a potential validity threat. We addressed this through both procedural and statistical remedies. Procedurally, we employed a time-lagged design to temporally separate the predictor (T1) from the outcome and moderators (T2), reducing transient mood effects and consistency motifs. We also assured anonymity and used reverse-coded items to minimize demand characteristics.

Statistically, we moved beyond the traditional Harman's single-factor test—which is often criticized for its insensitivity—and employed the more rigorous Unmeasured Latent Method Factor (ULMF) approach within the CFA framework (Podsakoff et al., 2003). We compared the model fit of our measurement model against a model where all items loaded on both their theoretical constructs and a latent common method factor. The results showed that adding the common method factor did not significantly improve model fit indices (ΔCFI < 0.01, ΔRMSEA < 0.01), and the factor loadings of the theoretical constructs remained significant. While these results suggest that CMB did not severely distort the structural relationships, we acknowledge that self-report limitations cannot be entirely eliminated by statistical means alone.

### 3.4 Ethical considerations

This study was conducted in strict accordance with the ethical principles outlined in the Declaration of Helsinki. Approval was obtained from the Institutional Review Board (IRB) of Qufu Normal University. All participants were informed about the study's purpose, procedures, potential risks and benefits, and their right to withdraw at any time without penalty. Confidentiality and anonymity were guaranteed; no personally identifiable information was collected. Informed consent was digitally obtained from all participants prior to their participation.

## 4 Results

This section details the statistical analyses performed to test the hypothesized moderated mediation model. The analysis proceeds in three stages: (1) preliminary analysis, including descriptive statistics, correlations, and common method bias assessment; (2) evaluation of the measurement model through Confirmatory Factor Analysis (CFA); and (3) testing of the structural model and specific hypotheses, including mediation, moderation, and moderated mediation, using path analysis with bootstrapping.

### 4.1 Preliminary analysis and common method bias

Before testing the main hypotheses, we examined the means, standard deviations (SD), and intercorrelations of the study variables. As shown in Table 1, all correlations were in the expected directions and statistically significant, providing initial support for our hypotheses. Notably, lower Need for Cognition (T1) was associated with a more positive affective experience (T1), higher Avoidance-Oriented GenAI Motivation (T2), and greater problematic use of Generative AI (T2). Furthermore, the moderators (Academic Stress and AI Literacy) were significantly correlated with the outcome variable, suggesting their potential relevance in the model.

Given that the data were collected via self-report measures, we took both procedural and statistical steps to address potential common method bias (CMB). Procedurally, the two-wave, time-lagged design temporally separated the measurement of the predictor/mediator variables from the outcome/moderator variables, which significantly reduces CMB (Podsakoff et al., 2003). Statistically, consistent with the approach outlined in the Methods section, we employed the Unmeasured Latent Method Factor (ULMF) approach using CFA. We compared the model fit of the theoretical measurement model against a model where all items loaded on both their theoretical constructs and a latent common method factor. The results indicated that the inclusion of the common method factor did not significantly improve model fit indices (ΔCFI < 0.01; ΔRMSEA < 0.01; ΔTLI < 0.01), and the factor loadings of the theoretical constructs remained significant and robust. These results suggest that common method bias does not substantially distort the hypothesized relationships in this study.

### 4.2 Measurement model

A Confirmatory Factor Analysis (CFA) was conducted in Mplus 8.3 to evaluate the six-factor measurement model, which included Need for Cognition, Positive Affective Experience, Avoidance-Oriented GenAI Motivation, Perceived Academic Stress, AI Literacy, and Problematic Use of Generative AI. The model demonstrated an excellent fit to the data: χ^2^ (549) = 1,125.6, *p* < 0.001; CFI = 0.96; TLI = 0.95; RMSEA = 0.048 [90% CI = 0.044, 0.052]; SRMR = 0.045. These indices meet the stringent criteria for good model fit (Hu and Bentler, 1999). Furthermore, the correlation matrix (Table 1) shows that the highest correlation between constructs is 0.56, which is well below the threshold of 0.90, providing further evidence that the constructs are distinct and not heavily inflated by method variance.

Convergent and discriminant validity were assessed. Convergent validity was supported as the Composite Reliability (CR) and Average Variance Extracted (AVE) for all constructs exceeded the recommended thresholds of 0.70 and 0.50, respectively (Need for Cognition: CR = 0.92, AVE = 0.58; Positive Affective Experience: CR = 0.89, AVE = 0.62; Avoidance-Oriented GenAI Motivation: CR = 0.86, AVE = 0.55; Problematic Use of GenAI: CR = 0.91, AVE = 0.63; Perceived Academic Stress: CR = 0.88, AVE = 0.60; AI Literacy: CR = 0.90, AVE = 0.61). Discriminant validity was established using the Fornell–Larcker criterion; as shown in Table 1, the square root of the AVE for each construct (the values on the diagonal) was greater than its correlation with any other latent construct. Collectively, the CFA results confirm that the measurement model is robust, providing a solid foundation for testing the structural model.

### 4.3 Structural model and hypothesis testing

The hypothesized moderated mediation model was tested using SEM in Mplus. The structural model included the main mediation pathway and the interaction terms between Avoidance-Oriented GenAI Motivation and the two moderators (Academic Stress, AI Literacy). The model fit was excellent: χ^2^ (556) = 1,245.8, *p* < 0.001; CFI = 0.95; TLI = 0.95; RMSEA = 0.052 [90% CI = 0.048, 0.056]; SRMR = 0.058. The model explained a substantial portion of the variance in Problematic Use of GenAI (*R*^2^ = 0.48).

#### 4.3.1 Main and mediation effects

First, we examined the paths of the core mediation model. The path from Need for Cognition (T1) to Positive Affective Experience (T1) was significant and negative (β = −0.41, *p* < 0.001), supporting H1. The path from Positive Affective Experience (T1) to Avoidance-Oriented GenAI Motivation (T2) was significant and positive (β = 0.45, *p* < 0.001), and the indirect effect of NFC on Avoidance Motivation via Positive Affect was significant (Effect = −0.18, 95% CI [−0.26, −0.11]), supporting H2. The path from Positive Affective Experience (T1) to Problematic Use (T2) was also significant (β = 0.25, *p* < 0.001). Finally, the path from Avoidance-Oriented GenAI Motivation (T2) to Problematic Use (T2) in the baseline model was significant (β = 0.30, *p* < 0.001).

Hypothesis 3 (mediation via Avoidance Motivation) and Hypothesis 4 (serial mediation) are best interpreted within the context of the full moderated mediation model below.

#### 4.3.2 Moderation effects

We then tested the moderation hypotheses (H5a and H5b). The interaction term between Avoidance-Oriented GenAI Motivation and Academic Stress was significant and positive (β = 0.15, *p* = 0.008), supporting H5a. The interaction term between Avoidance Learning Motivation and AI Literacy was significant and negative (β = −0.12, *p* = 0.021), supporting H5b.

**Figure 2:**
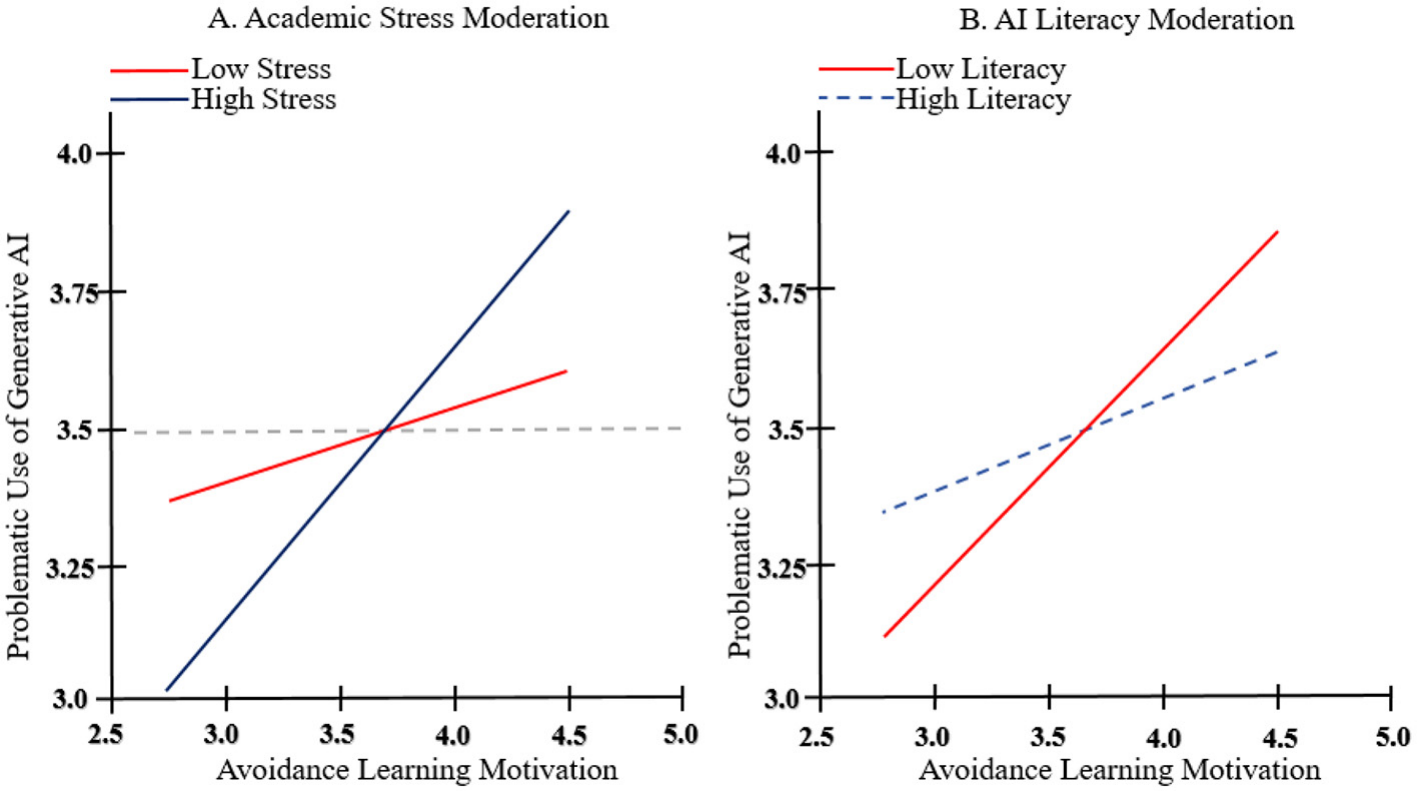
Dual moderation mechanisms: stress as risk amplifier vs. AI literacy as protective buffer in GenAI adoption. These plots illustrate the simple slope analyses for the significant moderation effects found in the structural model. **(A)** (academic stress): shows the relationship between avoidance-oriented GenAI motivation and problematic use at low (−1 SD) and high (+1 SD) levels of academic stress. The steeper slope for the “high stress” condition indicates that the link between avoidance motivation and problematic dependency is significantly amplified when students perceive high academic pressure. **(B)** (AI literacy): shows the relationship between avoidance-oriented GenAI motivation and problematic use at low (−1 SD) and high (+1 SD) levels of AI literacy. The flattened slope for the “high AI literacy” condition indicates a buffering effect, where high literacy effectively neutralizes the risk of dependency, whereas low literacy is associated with a strong positive relationship. All other variables in the model are held constant.

To interpret these interactions, we conducted simple slope analyses (Aiken and West, 1991). As illustrated in Figure 2, the positive relationship between Avoidance Learning Motivation and Problematic Use was stronger for students with high academic stress (β_high = 0.45, *p* < 0.001) compared to those with low academic stress (β_low = 0.15, *p* = 0.035). As shown in Figure 2, the relationship was stronger for students with low AI literacy (β_low = 0.42, *p* < 0.001) and became non-significant for those with high AI literacy (β_high = 0.18, *p* = 0.061), highlighting a powerful buffering effect.

#### 4.3.3 Moderated mediation effects

Finally, we tested the moderated mediation hypotheses (H6a and H6b) by examining the conditional indirect effects using the bootstrapping method (Table 2).

**Table 2:** Bootstrapping results for conditional indirect effects.

**Moderator**	**Level**	**Indirect effect**	**SE**	**95% bootstrap CI**	**Index of moderated mediation**
Academic stress	Low (−1 SD)	−0.028	0.015	[−0.059, −0.005]	−0.028
	High (+1 SD)	−0.083	0.022	[−0.131, −0.045]	[−0.051, −0.009]
AI literacy	Low (−1 SD)	−0.078	0.021	[−0.124, −0.041]	0.022
	High (+1 SD)	−0.033	0.018	[−0.070, 0.002]	[0.004, 0.045]

Indirect effect refers to the serial path from need for cognition -> positive affective experience -> avoidance-oriented GenAI motivation -> problematic use of GenAI. CI, confidence interval. The index of moderated mediation is significant if its 95% CI does not include zero.

For H6a, the serial indirect effect from Need for Cognition to Problematic Use (via Positive Affect and Avoidance Motivation) was significantly stronger at high levels of Academic Stress (Effect = −0.083, 95% CI [−0.131, −0.045]) than at low levels (Effect = −0.028, 95% CI [−0.059, −0.005]). The index of moderated mediation was significant (Index = −0.028, 95% CI [−0.051, −0.009]), providing strong support for H6a.

For H6b, the serial indirect effect was significant and negative for students with low AI Literacy (Effect = −0.078, 95% CI [−0.124, −0.041]), but was not significant for students with high AI Literacy (Effect = −0.033, 95% CI [−0.070, 0.002]). The index of moderated mediation was significant (Index = 0.022, 95% CI [0.004, 0.045]), supporting H6b.

These results collectively confirm our full model. The findings provide support for the moderating roles of academic stress and AI literacy (H5a and H5b) and, by extension, the underlying serial mediation pathway (H3 and H4), which is shown to be conditional (H6a and H6b).

## 5 Discussion

This study investigated the psychological pathway leading to problematic use of Generative AI by testing a moderated mediation model grounded in the I-PACE framework. Our findings offer a nuanced account of how a predisposing cognitive style, mediated by specific affective-cognitive responses, translates into digital dependency, and how this process is bounded by both situational pressures and personal resources.

### 5.1 General discussion of key findings

#### 5.1.1 The pathway to dependency: from low cognitive effort to avoidance

Consistent with our primary hypotheses (H1–H4), our results confirmed the proposed serial mediation chain. Students with a lower Need for Cognition—a preference for less effortful thinking—were more likely to experience a strong positive affective response when using GenAI. This “quick-fix” satisfaction, in turn, fostered an avoidance-oriented learning motivation, which ultimately was associated with problematic use.

This finding makes a significant contribution by specifying the affective-motivational mechanism through which a general cognitive trait is linked to a specific problematic behavior. It extends the I-PACE model by demonstrating that for instrumental technologies like GenAI, the initial gratification is not merely hedonic pleasure but is deeply intertwined with cognitive relief and the subsequent development of a maladaptive learning strategy. The positive feeling is not just about fun; it is about the rewarding experience of circumventing cognitive struggle. This establishes “avoidance-oriented genAI motivation” as a critical, and previously underexplored, cognitive mediator in the context of problematic technology use.

#### 5.1.2 The role of moderators: when the pathway accelerates or halts

Perhaps the most crucial contribution of our study lies in the identification of boundary conditions that govern this psychological pathway (H5a, H5b). Our model revealed that the link between an established avoidance motivation and actual problematic use is not deterministic but is powerfully shaped by context and personal competence.

Specifically, we found that high academic stress acts as an “accelerant.” When students who are already motivated to avoid challenges face intense academic pressure, their reliance on GenAI escalates from a preference to a perceived necessity. This aligns perfectly with the Conservation of Resources (COR) theory (Hobfoll, 1989). Under stress, individuals prioritize resource preservation, and GenAI offers a low-effort strategy to meet academic demands, thus conserving cognitive energy. Our findings empirically demonstrate how a situational stressor can transform a latent motivation into manifest problematic behavior, highlighting a vulnerable population: students who are both motivationally at-risk and environmentally pressured.

Conversely, our results showed that AI literacy acts as a “brake” or a protective buffer. For students with high AI literacy, the link between avoidance motivation and problematic use was significantly weakened, even becoming non-significant. This is a novel and vital finding. It suggests that knowledge and critical thinking about the technology itself can empower individuals to self-regulate. Literate users, even if tempted to avoid effort, possess the metacognitive skills to recognize the limitations of AI, question its outputs, and understand the long-term detriments of over-reliance. They can maintain a functional, rather than dysfunctional, relationship with the tool. This finding moves beyond a simplistic “technology is bad” narrative to a more sophisticated understanding where user competence is a key determinant of outcomes.

Furthermore, these findings underscore the necessity of operationalizing AI literacy not merely as a technical skill but as a set of teachable competencies embedded within course design. To effectively buffer against problematic reliance, educators should cultivate environments that prioritize critical engagement over prohibition, such as establishing clear transparency norms, integrating explicit learning outcomes for critical AI evaluation, and utilizing reflective logs where students document their human-AI collaboration process. Activities that challenge students to critique AI bias and hallucinations can transform the tool from an “answer machine” into an object of inquiry. This aligns with recent scholarship suggesting that academic integrity is best supported not by deterrence alone, but by designing educational ecosystems that actively build digital literacy and critical thinking, thereby reducing the psychological need for dishonest shortcuts (Tan and Maravilla, 2024).

#### 5.1.3 The conditional nature of digital dependency

By confirming the moderated mediation hypotheses (H6a, H6b), our study provides a holistic picture of the phenomenon. The entire indirect pathway from a low-effort cognitive style to problematic use is conditional. This means that the risk posed by a low Need for Cognition is not uniform; it is most potent for students who are under high academic stress and possess low AI literacy. Conversely, the risk is substantially mitigated for students with high AI literacy, even if they are experiencing stress. This interaction underscores the complex interplay between person, behavior, and environment in the development of digital dependency.

### 5.2 Theoretical implications

This research offers several significant theoretical contributions that advance our understanding of digital dependency in the context of instrumental technologies.

First, and most fundamentally, this study contributes to the literature by establishing a clear theoretical conceptualization of “Problematic GenAI Use” and extending the I-PACE model to instrumental technologies. While previous research has often conflated excessive technology use with clinical addiction, our findings support a more nuanced perspective rooted in the cognitive-behavioral framework. We demonstrate that problematic GenAI use should not be viewed merely as a clinical dependency, but rather as a maladaptive functional coping style. This distinction clarifies that the core issue is not physiological addiction, but a psychological over-reliance on GenAI to regulate negative affect and avoid cognitive effort. Based on this conceptualization, our study empirically validates the extension of the I-PACE framework—originally conceived for hedonic applications like gaming—to utilitarian tools like GenAI. We specify a unique affective-cognitive pathway—”cognitive relief leading to avoidance motivation”—that is particularly salient for technologies designed to reduce cognitive load. This finding enriches the model by demonstrating that the core reinforcing experience can be utilitarian and cognitive in nature, rather than purely hedonic. It is important to clarify that while our definition of problematic use focuses on psychological dependency and the inability to self-regulate, it inevitably overlaps with integrity concerns. As noted by Seran et al. (2025), the cognitive dissonance resulting from this dependency often leads to rationalizations that can justify academic misconduct; however, our construct primarily captures the compulsive reliance itself rather than the specific act of cheating. Crucially, this shifts the theoretical focus from “pleasure-seeking” to “cognitive-strain-avoidance” as a primary driver of dependency. This mechanism differs from the traditional focus on craving for emotional rewards, suggesting that problematic use of instrumental tools may be better characterized as a form of “cognitive dependency” rather than a classic behavioral addiction driven by impulsivity.

Second, this study integrates stress-and-coping theories with a model of digital dependency by empirically demonstrating the “accelerant” role of academic stress. The finding that stress amplifies the path from avoidance motivation to problematic use provides strong support for a stress-vulnerability perspective in digital health. It bridges the literature on academic burnout and problematic technology use, showing how the former can be a direct catalyst for the latter. This suggests that in high-pressure environments, GenAI is not just a tool but becomes a maladaptive coping mechanism, consistent with Lazarus and Folkman's (1984) transactional model of stress.

Third, and perhaps most innovatively, our research introduces a non-pathological, competence-based variable—AI literacy—as a key protective factor, thereby enriching the “Person” component of the I-PACE model with a dynamic, malleable resource. Much of the literature on problematic technology use focuses on stable, often pathological, risk factors. By demonstrating that AI literacy acts as a powerful “brake,” effectively neutralizing the negative pathway even in the presence of an avoidance motivation, our study opens up new avenues for strengths-based research. This finding highlights the agentic role of the user, suggesting that “executive control”—a core I-PACE component—is not just about inhibiting impulses but can be bolstered by specific digital competencies. It shifts the scholarly conversation from “what is wrong with the user” to “what skills and competencies can protect the user,” framing self-regulation as a learnable skill in the digital age.

Fourth, while our model focuses on avoidance motivation as a proximal mediator, Self-Determination Theory (SDT) offers a complementary “upstream” lens for understanding why students gravitate toward such cognitive relief. As Tan and Maravilla (2024) argue, academic integrity and intrinsic motivation flourish when students' basic psychological needs for autonomy, competence, and relatedness are met. From this perspective, the “Avoidance-Oriented GenAI Motivation” observed in our study likely arises when these needs are thwarted—specifically, when students feel incompetent to meet high demands or lack autonomy in their learning process. Consequently, AI literacy interventions act as a buffer not only by providing skills but by restoring a sense of competence and autonomy, thereby disrupting the psychological impulse to seek effortless shortcuts.

### 5.3 Practical implications

The findings have direct and actionable implications for educators, university administrators, and students.

Shift from Prohibition to Education: Instead of banning GenAI tools, educational institutions should focus on cultivating critical AI literacy. Workshops and curriculum modules should be designed to teach students not just how to use these tools, but how to critically evaluate their outputs, understand their limitations, and use them ethically as a supplement, not a substitute, for their own thinking. Our results strongly suggest that this is the most effective way to prevent problematic use.

Targeted Support for At-Risk Students: Our model identifies a clear at-risk profile: students with a low tendency for effortful cognition who are under high academic stress. University counseling and academic support centers can use this information to provide targeted interventions. This could include workshops on stress management and healthy coping strategies, as well as academic coaching that encourages deep learning over surface-level, avoidance-based tactics.

Rethinking Pedagogy and Assessment: Educators should move beyond the futile “arms race” of AI detection toward designing assessments that value the process of learning over the final product. Consistent with recent calls to shift from recall-based tasks to authentic demonstrations of thinking (Tan and Maravilla, 2024), we recommend incorporating process documentation, where students must submit version histories or reflective logs detailing how they developed their ideas. Furthermore, introducing interactive oral defenses or in-class validation tasks can effectively verify that the submitted work reflects the student's own understanding. By assigning authentic, context-specific problems that require personal synthesis rather than generic answers, educators can inherently promote a higher Need for Cognition and discourage the avoidance-oriented approach that GenAI facilitates.

Reflective Pedagogy for Dissonance Reduction: Finally, to address the cognitive dissonance that often drives avoidance behaviors, we recommend implementing reflective pedagogical interventions. As suggested by Seran et al. (2025), constructivist approaches such as requiring students to maintain “process journals” of their AI use, write justification statements for AI-generated inclusions, or compare AI-generated drafts with their own writing can make the dissonance explicit. These practices force students to confront the gap between their effort and the output, turning a potential source of anxiety and avoidance into a conscious learning moment that reinforces autonomy rather than dependency.

### 5.4 Limitations and future research

Despite its strengths, this study has several limitations that suggest directions for future research.

First, regarding the research design, although we employed a time-lagged approach to separate the predictor (Need for Cognition at T1) from the outcome (Problematic Use at T2), a limitation remains regarding the temporal ordering of the final stage. Specifically, the mediator (Avoidance-Oriented GenAI Motivation), the moderators (academic stress, AI literacy), and the outcome variable were all measured concurrently at Time 2. This concurrent measurement limits our ability to make strict causal inferences, particularly regarding the directionality of the moderation effects and the link between motivation and problematic use. While the theoretical framework supports a directional pathway, the relationships observed among T2 variables should be interpreted as associations consistent with the proposed model rather than definitive directional evidence. Future research should employ multi-wave longitudinal designs (with three or more waves) or experimental manipulations to rigorously verify these links.

Second, our sample was drawn from a single university in China operating under a “permissive but cautious” AI policy. This specific regulatory context limits generalizability. In institutions with strict bans, the relationship between stress and problematic use might be stronger due to the added anxiety of secrecy. Conversely, in environments with full AI integration and training, the protective effect of AI literacy might be more pronounced. Future studies should replicate this model across diverse cultural and institutional contexts to examine how differing AI governance regimes alter these observed relationships.

Third, a major limitation of this study lies in its exclusive reliance on self-report measures. While we employed statistical controls for common method bias, self-reports regarding “problematic use” can be influenced by subjective perception or denial. To robustly validate the construct of problematic dependency, future research is strongly encouraged to incorporate objective behavioral or system-level data, such as interaction logs, prompt frequency analysis, and session duration metrics, alongside self-reports.

Finally, we acknowledge a potential for conceptual overlap between Avoidance-Oriented GenAI Motivation and Problematic Use, as both measures reference GenAI behaviors. Although our CFA results supported discriminant validity, future work could operationalize avoidance motivation in a more technology-general manner (e.g., general academic avoidance) to further disentangle the motivation from the specific tool used to satisfy it. By doing so, researchers can better isolate the psychological intent to avoid effort from the behavioral pathology of dependency.

## 6 Conclusion

This study delineated a psychological map of students' progression from cognitive convenience to digital dependence in the age of Generative AI. We found that a low-effort cognitive style fosters an over-reliance on AI, not directly, but through a reinforcing cycle of cognitive relief and learned avoidance of academic struggle. Crucially, this pathway is not deterministic; its potency is amplified by academic stress yet buffered by the user's AI literacy.

The central thesis emerging from our findings is that the antidote to the challenges posed by AI lies not in technological prohibition but in the cultivation of human agency and digital wisdom. AI literacy, conceptualized here beyond mere technical skill, represents a core psychological resource—a form of “intellectual immunity” that empowers individuals to maintain critical autonomy in a technologically saturated environment. It is this capacity for self-regulation, rather than the absence of temptation, that ultimately distinguishes beneficial use from problematic dependence.

As we move deeper into an era of human-AI symbiosis, future research and educational policy must pivot from a narrow focus on the technology itself to a more ecological perspective. Understanding the dynamic interplay between the individual, their environment, and the tools they use is paramount. Only by fostering resilient, critical, and literate users can we ensure that generative technologies serve as instruments of human augmentation, not as architects of intellectual passivity.

## Data Availability

The data analyzed in this study is subject to the following licenses/restrictions: the data presented in this study are not publicly available due to privacy and ethical concerns regarding the human participants involved. Qualified researchers may request access to the data by contacting the corresponding author. Requests to access these datasets should be directed to Tongqiang Dong, qfnudtq@qfnu.edu.cn.

## Appendix A

Scale Items

1. Avoidance-Oriented GenAI Motivation Adapted from the performance-avoidance goals subscale of the Achievement Goal Questionnaire-Revised (Elliot and Murayama, 2008). Items were contextualized to specifically measure the intent to offload cognitive effort viaGenAI.
  - My main goal when using GenAI for my studies is to avoid difficult tasks as much as possible.
  - I often use GenAI to escape the pressure of challenging academic assignments.
  - When I encounter a hard problem, I immediately turn to GenAI so I don't have to struggle with it myself.
  - I rely on GenAI to complete my work quickly specifically to avoid the effort of deep thinking.
2. AI Literacy Developed based on the conceptual framework by Long and Magerko (2020), specifically targeting “critical evaluation” and “ethics”. (7items)
  - I am good at critically judging whether the information provided by Generative AI is accurate and reliable.
  - I can identify potential biases or stereotypes in the content generated by AI.
  - I understand the ethical implications (e.g., copyright, academic integrity) of using Generative AI.
  - I am able to evaluate the limitations of Generative AI when applying it to my coursework.
  - I know how to verify the sources of information provided by Generative AI models.
  - I can distinguish between tasks that require human judgment and those that can be safely delegated to AI.
  - I am aware of how Generative AI models might “hallucinate” or fabricate incorrect facts.
